# Supplementary material for: Fexinidazole and Corallopyronin A target Wolbachia-infected sheath cells present in filarial nematodes
Source: bioRxiv. 2025 Jan 26:2025.01.23.634442. Preprint. [Version 1] doi: 10.1101/2025.01.23.634442 (PMC11785234; doi:10.1101/2025.01.23.634442)
Supplement: Supplement 1 [file NIHPP2025.01.23.634442v1-supplement-1.pdf]

Supplemental Information

Supplemental Figure 1. *Wolbachia*-infected sheath cells are also present in Brugia malayi ovarian tissues.

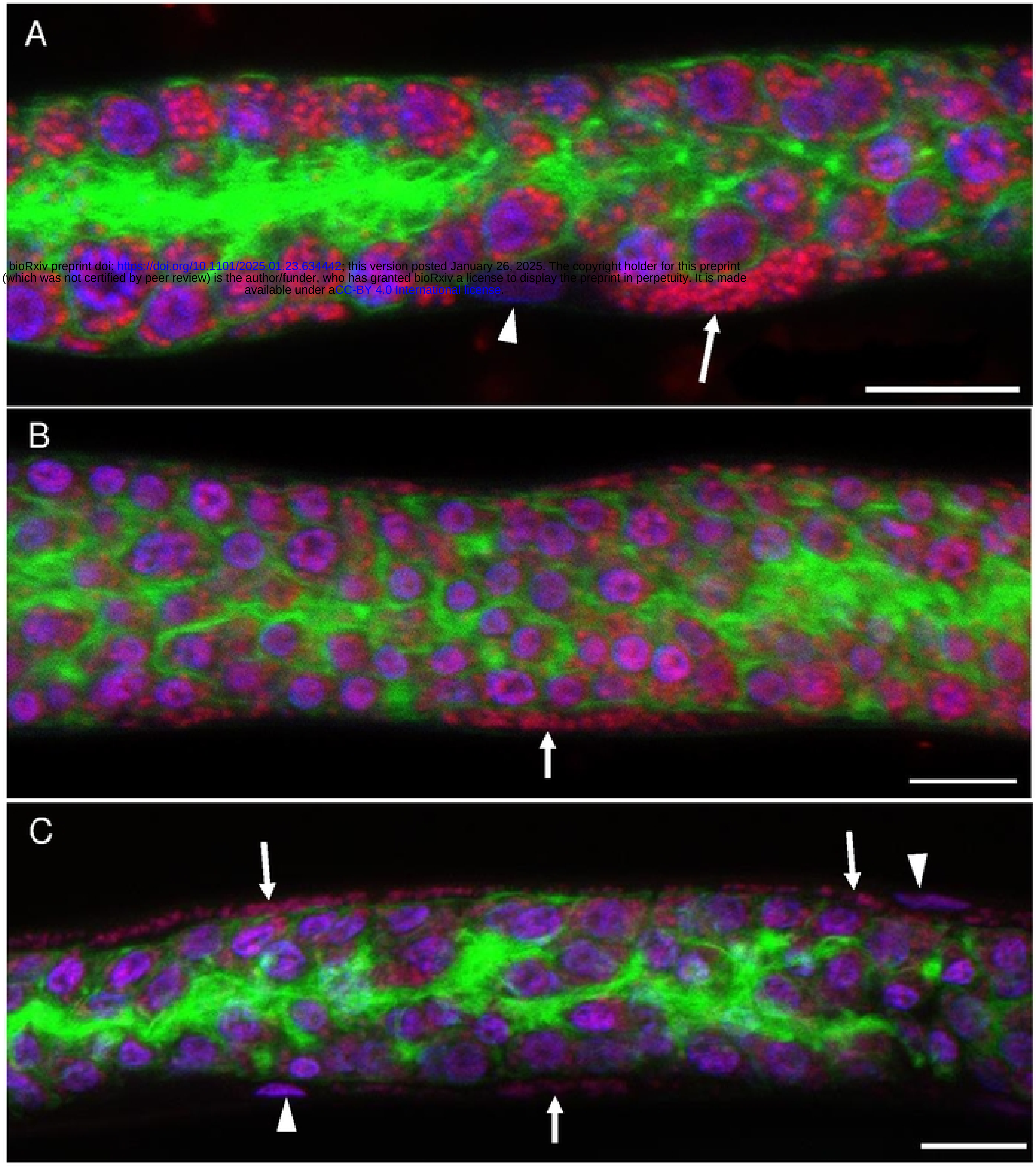

bioRxiv preprint doi: https://doi.org/10.1101/2025.01.23.634442; this version posted January 26, 2025. The copyright holder for this preprint (which was not certified by peer review) is the author/funder, who has granted bioRxiv a license to display the preprint in perpetuity. It is made available under aCC-BY 4.0 International license.

Supplemental Figure 1. Wolbachia-infected sheath cells are also present in Brugia malayi ovarian tissues. A-C) Wolbachia clusters are found in one of the species of filarial nematode that infects humans, Brugia malayi. Nematode germline tissue is stained with Propidium Iodide (red), DAPI (purple), and Phalloidin 488 (green). White arrows point to Wolbachia clusters in infected sheath cells. White arrowheads point to sheath cell nuclei. All scale bars are 10µm.

Supplemental Figure 2. Wolbachia in the infected sheath cells do not incorporate EdU after 72 hours.

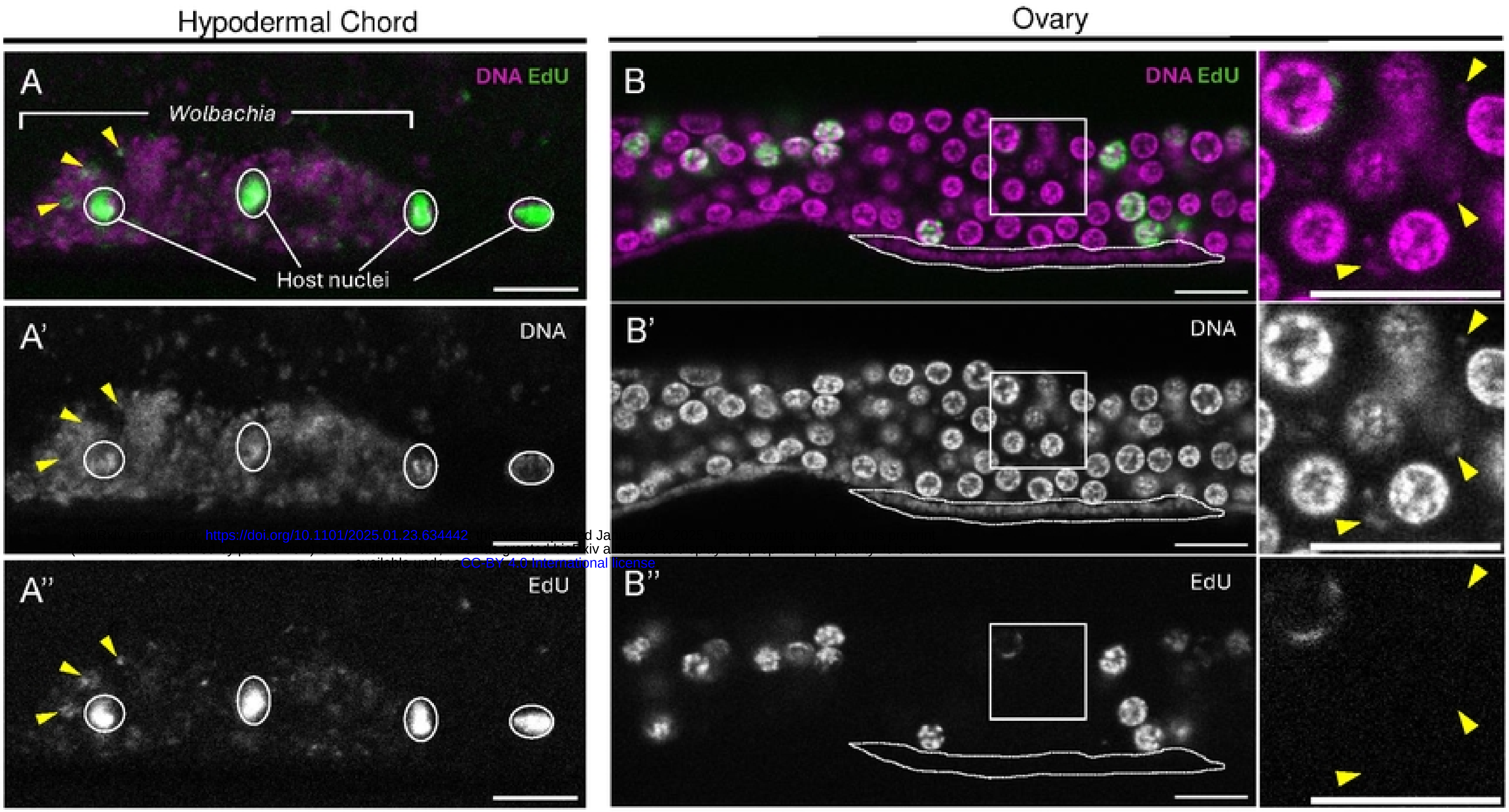

[illegible] https://doi.org/10.1101/2025.01.23.634442 [illegible]
available under a CC-BY 4.0 International license

Supplemental Figure 2. Wolbachia in the infected sheath cells do not incorporate EdU after 72 hours.

A-A'') Hypodermal chords of adult Brugia pahangi were incubated with 200 µM EdU for 72 hours. Nematode host nuclei are outlined in white. All other magenta puncta are Wolbachia. EdU incorporation can be seen amongst the Wolbachia puncta. Image represents a max projection of four z-stacks with a step size of 0.38 µm. DNA is stained with DAPI only. **B-B'')** Ovarian tissue of adult Brugia pahangi was incubated with 200 µM EdU for 72 hours. EdU does not incorporate in Wolbachia-infected sheath cells (white dotted outline; a total of 7 infected sheath cells were analyzed). The boxed region is enlarged in the inset to the right. Nematode host oocyte nuclei incorporate EdU, but Wolbachia puncta do not (yellow arrowheads point to three representative Wolbachia puncta). DNA is stained with DAPI only. For all images, EdU is visualized with Invitrogen Click-iT EdU imaging kit, Alexa Fluor 488. All scale bars are 10 µm.

Supplemental Table 1. List of repurposed drugs screened for anti-wolbachial activity in infected sheath cells.

Drug	Mechanism/Target	Reference
Albendazole	Nematode beta-tubulin	Borgers, et al. 1975
Albendazole sulfone	Nematode beta-tubulin	Marriner, et al. 1980
Albendazole sulfoxide	Nematode beta-tubulin	Marriner, et al. 1980
Colistin sulfate	Phospholipid A, outer membrane	Velkov, et al. 2013
Corallopyronin A	DNA-dependent RNA polymerase	Krome, et al. 2022
Doxycycline	Bacterial 30S ribosomal subunit	Nguyen, et al. 2014
Fexinidazole	Prodrug: nitroreductase-activated	Deeks, 2019
Metronidazole	Prodrug: oxidoreductase-activated	Dingsdag and Hunter, 2018
Pararosaniline pamoate	Heat Shock Protein 90	Shahinas, et al. 2015
Rapamycin	mTOR	Ballou and Lin, 2008
Rifampicin	DNA-dependent RNA polymerase	Mosaei, et al. 2019

bioRxiv preprint doi: https://doi.org/10.1101/2025.01.23.634442; this version posted January 26, 2025. The copyright holder for this preprint (which was not certified by peer review) is the author/funder, who has granted bioRxiv a license to display the preprint in perpetuity. It is made available under aCC-BY 4.0 International license.

Supplemental Table 1. List of repurposed drugs screened for anti-wolbachial activity in infected sheath cells.

Supplemental Figure 3. The nitroreductase gene (ntr) is expressed by *Wolbachia* in the nematode germline.

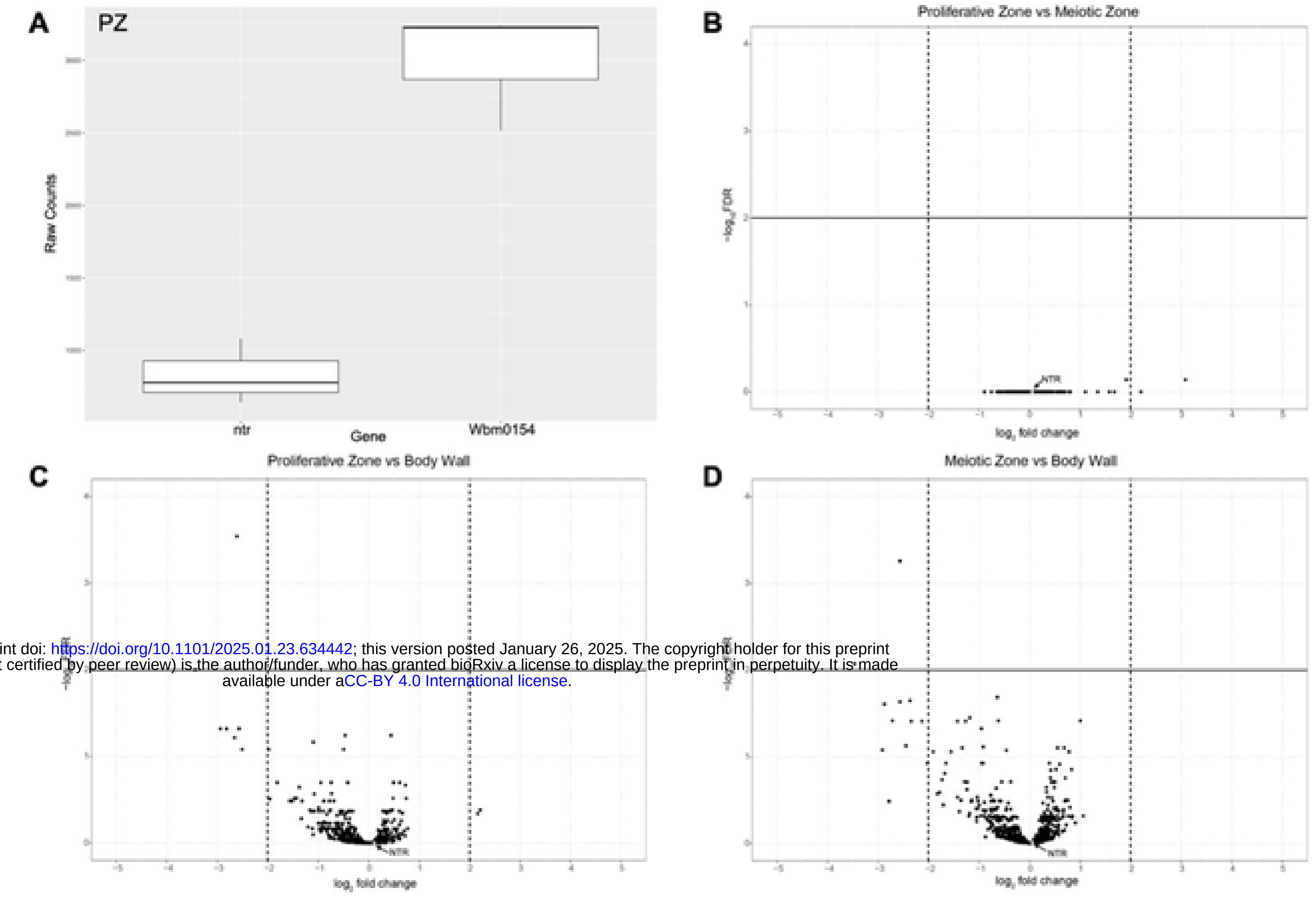

bioRxiv preprint doi: https://doi.org/10.1101/2025.01.23.634442; this version posted January 26, 2025. The copyright holder for this preprint (which was not certified by peer review) is the author/funder, who has granted bioRxiv a license to display the preprint in perpetuity. It is made available under aCC-BY 4.0 International license.

Strain	Nitroreductase protein length	Gene accession	Protein sequence
Wolbachia endosymbiont of Brugia malayi wBm	149	WP_011256715.1	MISKEDLLELMKIRHSRRSYGQSKLAHQEEINMLIETAWLSPSRYGDEPWRQVICNRQSNQDA<br>WEKLLSCPTEPNQKWAKDTQILIISLSTKNFHDHTKIVNFWGSHDTGIANYTFMLQSYIYEFNAH<br>QMSRFDRNKIVKNSIYQMILI
Wolbachia endosymbiont of Brugia pahangi wBp	149	WP_246166039.1	MISKEDLLELMKIRHSRRSYGQSKLAHQEEINMLIETAWLSPSRYGDEPWRQVICNRQSNQDA<br>WEKLLSCPTEPNKKWAKDTQMLIISLSTKNFHDHTIKSKFLGQPRYWCSKLYIYATGYIYEFNAH<br>QMSRFDRNKIVKNSIYQMILI
Wolbachia endosymbiont of Cruorifilaria tuberocauda	187	QKX01752.1	MINTQDLLALMRIRHSGCLYDPNKVVNQEKINLLIEAARLSPSCFGDEPWRYIICNKQSNQNSW<br>VKLLNCLDESNQKWAKNAQVLIISLSAKNFRKLDKGKNFWAKHDTGAANYALILQAASANLMGH<br>QVGGFDGNKIAKKFNIPSDFNITSVIAVGYEEEGTKVNEKKRRPVEEIFFYDEWPSELI
Wolbachia endosymbiont of Dipetalonema caudispina	187	QKX01080.1	MINTQDLLALMRTRHSGCLYNPNKLVNQEEINLLIEAARLSPSCFGDEPWRYIICNKQNNQNSW<br>EKLLSCLDKSNQKWAKNAQILIISLSAKNFRELNKGKNFWAKHDTGAANYALMLQAASINLMAH<br>QVGGFNRNQIVKKFNIPNGFNITSVIAVGYEEEGAEVKEKKRRPIEEIFFYDEWPGEFI
Wolbachia endosymbiont of Dirofilaria immitis	185	WP_175818410.1	MMNTQDLLALMKIRHSGCLYDPDKVVNQEKINALIEAARLSPSCFGDEPWRYIICNKQSNQNSW<br>KKLLSCLDESNQKWAKKAQVLIISLSAKNFRKLDKGKNFWAKHDTGAANYALMLQAASTNLMAH<br>QVGGFDRNKIVKRFNIPNDF NITSVIAVGYEEEGTEVQEKKRRPIEEIFFYDEWPLS
Wolbachia endosymbiont of Litomosoides sigmodontis	185	QKX02732.1	MISKEDLLELIKIRHSGRSYDQSKLVHQKEINMLIEATRLSPSCYGDEPWRYVICNRQSNQNAWK<br>KLLSCLTEYNQKWTKDTQILIISLSAKNFRDHTKGANFWGSHDTGAANYALMLQATSMNLMAHQ<br>MGGFD RDKIVKKFNIPDDFNVMSVIAIGYEKKGAEVKEKTRKPIEEIFFYNEWPKS
Wolbachia endosymbiont of Onchocerca ochengi	187	CCF78436.1	MVNTQDLLTLMKIRHSGCLYDPDKVVNQEEINSLIEAARLSPSCFGDEPWRYVICNKQSNQSSW<br>KKLLSCLNESNQKWAKNAQVLIISLSAKNFRELDKGENFWAKHDTGAANYALMLQAASTNLMAH<br>QVGGFDRNKIVKKFNIPNDFNITSVIAVGYEEEGAEIKEKKRRPVEEIFFYDEWPSELT
Wolbachia endosymbiont of Onchocerca volvulus	172	NZ_HG810405.1:4<br>74294-474856	MVNTQDLLTLMKIRHSGCLYDPDKVVNQEEINSLIEAARLSPSCFGDEPWRYVIYNKQSNQSSW<br>KKLLSCLNESNQKWAKNAQVLIISLSAKNFRELDRGENFWAKHDTGAANYALMLQAASTNLMAH<br>QVGGFDRNKIVKKFNIPNDFNITSVIAVGYEEEGAEIKEKKEGQ<br>(Frameshifted; NCBI annotated as pseudogene)
Wolbachia endosymbiont of Wuchereria bancrofti	129	OWZ25411.1	MISKEDLLELMKIRHSRHSYGQSKLAHQEEINMLIETARLSPSRYGDEPWRHVICNRQSNQDAW<br>EKLLSCPTEPNQKWAKDTQISTISLSAKNFHDHTKRANFWGSHDIGAANYTLTLQATSMNLMLIR<br>(Frameshifted; premature stop)

Supplemental Figure 3. The nitroreductase gene (ntr) is expressed by Wolbachia in the nematode germline. The nitroreductase gene (ntr) is expressed at similar levels by wBm in three B. malayi host tissues: the proliferative zone (PZ), the meiotic zone (MZ), and the body wall (BW). **A)** Raw counts of ntr and actin-like gene Wbm0154 output by featureCounts for the PZ dissection. While technical aspects can affect count number, this illustrates that the ntr gene is expressed, but at a lower level relative to actin-like Wbm0154. **B-D)** Volcano plots showing similar expression of ntr across the three tissues. The Y axis is -$\log_{10}$ false discovery rate (FDR) and the X axis is $\log_2$ (fold change). The ntr gene is denoted in each plot. Sequencing library data were obtained from Chevignon et al. (2021). The solid black horizontal line and the vertical dashed lines denote the criteria used in Chevignon et al. (2021) for their assessment of differential gene expression: $|\log_2(\text{fold change})|>2$ with an FDR <0.01. The ntr gene, like the majority in the analysis of Chevignon et al. (2021), is not differentially expressed between tissues. The relative expression of the nitroreductase is similar to expression of several other genes that include nusB (a transcription termination factor), ribosomal protein L17, and tRNA-Thr. Table indicates conserved nitroreductase genes found in the genome sequences of nine filarial nematode species.
